# Supplementary material for: The Ontogeny and Dietary Differences in Queen and Worker Castes of Honey Bee (Apis cerana cerana)
Source: Insects. 2024 Oct 31;15(11):855. doi: 10.3390/insects15110855 (PMC11595054; doi:10.3390/insects15110855)
Supplement: Supplementary file 1 [file insects-15-00855-s001.zip › insects-3202758-supplementary.pdf]

Table S1. Moisture (%, fresh weight) and crude protein (%, dry weight) content of RJ and WJ at different days of *Apis cerana cerana.* The data are reported as means ± SE (n=3). An asterisk (*) next to the higher value of RJ or WJ indicates a significant difference between the RJ and WJ samples on the corresponding day at P <0.05. Different lowercase letters (a, b, c) in a column indicate significant differences among RJ or WJ samples at P <0.05.

Type		1d	2d	3d	4d	5d
Moisture	WJ	68.52b	72.06ab*	76.55a*	70.26b	58.54c
	RJ	68.52a	57.51c	63.52b	66.00ab	58.09c
Crude protein	WJ	45.62b	48.13ab	54.85a	46.84ab	26.56c
	RJ	45.62b	51.46a	51.97a	49.48ab	40.41c*
